# Photoexcited Intramolecular Charge Transfer in Dye Sensitizers: Predictive In Silico Screening for Dye-Sensitized Solar Cell Devices

**DOI:** 10.1021/acsomega.1c06233

**Published:** 2022-04-13

**Authors:** Kalyani Chordiya, Md. Ehesan Ali, Mousumi U. Kahaly

**Affiliations:** † ELI-ALPS, ELI-HU Non-Profit Ltd., Wolfgang Sandner utca 3, Szeged H-6728, Hungary; ‡ Institute of Physics, University of Szeged, Dóm tér 9, H-6720 Szeged, Hungary; ¶ Institute of Nano Science and Technology, Mohali, Punjab 140306, India

## Abstract

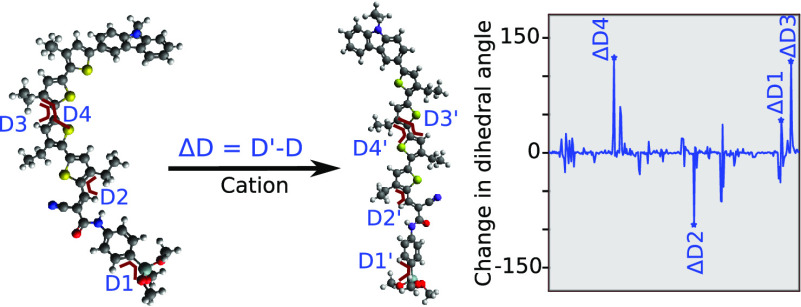

Efficient photoinduced intramolecular charge transfer (ICT) from donor to acceptor in dye molecules is the functional basis and key property in the working of a dye-sensitized solar cell (DSSC). To understand the ICT process in photoexcited dye molecules, we analyze the electronic properties and structural parameters of a chosen set of experimentally synthesized donor–acceptor (D–A) and donor−π-spacer−acceptor (D−π–A) type dye molecules in their ground, excited, and cationic states. The correlation between structural modification and charge redistribution in different parts of the molecule helps to identify the extent of π-conjugation and spatial rearrangement of electron density localization along the molecular skeleton. We find that prominent twisting of several groups and the resulting molecular bond rearrangements in larger parts of the molecule promote efficient donor to acceptor ICT, such as in D–A type ADEKA1 and C275 dyes. Thus, based on the modest computation of structural and electronic properties of dye molecules in their respective ground, excited, and cationic states, we identify the desired structural changes that facilitate tunable intramolecular charge transfer to highlight a simple and direct prescription to screen out probable efficient dye molecules among many samples. Our approach complements recent experimental evidence of capturing the structural view of the excited-state charge transfer in molecules.

## Introduction

**Figure 1:**
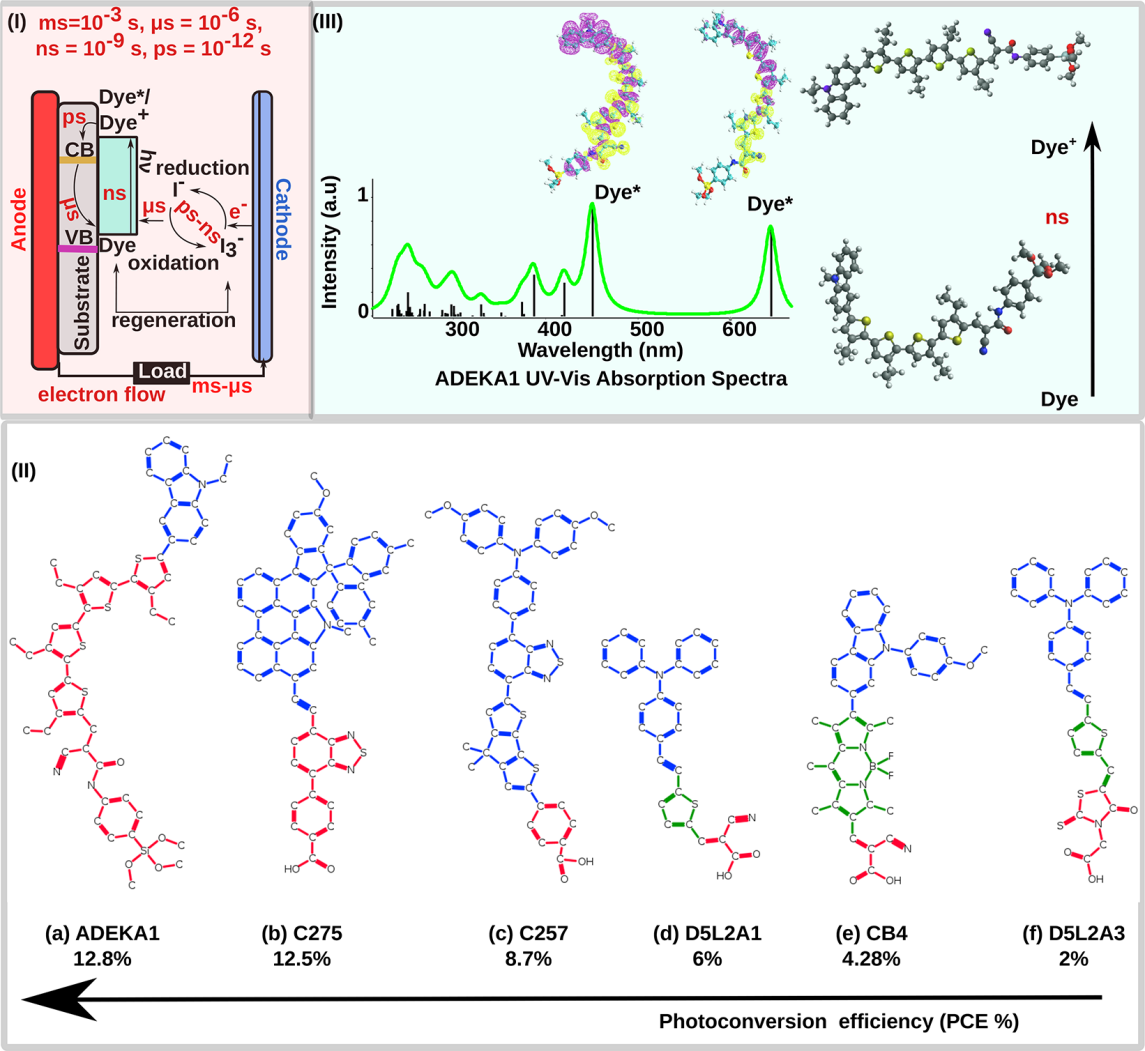
(I) Schematics of a DSSC with the electron transfer process involved shown by black one-direction arrows and the time scale for the electron transfer. (II) 2-D structural representation (PCE %) of dyes: (a) ADEKA1 (12.8%), (b) C275 (12.5%), (c) C257 (8.7%), (d) D5L2A1 (6%), (e) CB4 (4.28%), and (f) D5L2A3 (2%). The blue color represents the donor group, green the π-spacer linkage, and red the acceptor. Note that there are ambivalent opinions about such distinction of the D, π, and A parts in dyes like ADEKA-1 or C275, and the present colored representations are just representations of descriptions in the mentioned references. (III) UV–vis spectra (green curve) of ADEKA1 spanning the spectral range of 220–700 nm, suggesting adequate light absorption, along with electron charge density relocalization in the excited Dye (Dye*) corresponding to two peaks in the UV–vis spectra. The Dye* isosurface shows the electron density upon the photoexcitation of the dye with a particular frequency. The right panel shows the structure or geometry change owing to the Dye to Dye*/Dye^+^ transition, which occurs within a nanosecond time scale.^15^

Dye-sensitized solar cells (DSSCs) were initiated after their first report by O’Regan and Grätzel in 1991,^1−3^ and with this rapid development was observed in the DSSC owing to their low-cost materials,^4^ flexible designs,^5^ high performance, and stability under low- or diffuse light conditions.^6^ The research efforts are mainly focused on tailoring the efficiency and stability by designing new sensitizers,^7−9^ modifying the semiconductor band gap,^10,11^ and optimizing the redox couple,^12^ dye absorbance,^13^ and counter electrodes.^14^ The typical working process of the device starts with photoexcitation of the dye molecule (from Dye → Dye*/Dye^+^ in nanoseconds). The excited dye molecule (Dye*) will release an electron into the conduction band of the semiconductor material (on a picosecond time scale), leaving the dye in the cationic (Dye^+^) state.^15^ The released electrons from the anode move through an external circuit and reach the cathode in 10^–8^–10^–1^ s.^16^ Then the oxidized dye is regenerated when the dye receives electrons from a redox mediator (I^–^/I^–3^) (μs). Oxidation of the medium happens in the process within picoseconds to nanoseconds. Further, these oxidized redox mediators (I^–3^) diffuse to the counter electrode, where it is regenerated by the electrons reaching the counter electrode through an external circuit. Thus, the dye and the electrolyte solution regain the original state and are ready to repeat the cycle. The working schematics of a typical DSSC with time scales involved in the electron transfer cycle of the device are given in Figure 1(I). Thus, it is obvious that the efficiency of the device is highly sensitive to the structure and performance of the dye molecules.^17^

The dye molecules typically of the donor–acceptor (D–A) and donor–pi–acceptor (D−π–A) type have attracted immense attention in the field of DSSCs.^18^ The electron-donating groups with aromatic amines such as triphenyl amine,^19^ carbazole,^20^ and triazatruxene^21^ owing to their high positive mesomeric effects, synthetic availability, and modularity are best suited as donor groups. The π-spacer links the donor and acceptor group and has to have the electron affinity higher compared to the donor and lower than the acceptor group.^22^ Upon photoexcitation of the dye, the electron density of this molecule is rearranged and trapped by the highly electronegative groups such as π-spacer and acceptor groups.^23^

The SM-315 dye reported by Mathew et al. with 13%^24^ power conversion efficiency (PCE) and ZL001 and ZL003 by Zhang et al. with 12.8% and 13.6% PCE, respectively,^21^ are considered highly efficient D– π–A dyes. To improve the efficiency further, there were variations in the structure such as D–A, D−π–π–A, D–A′−π–A, and D–D−π–A:^25−27^ changes in the donor in order to extend the π-conjugation,^7^ π-spacer bearing a strong electron donor group and high molar absorption coefficient,^8,28^ and acceptor and linker as a stronger electron-withdrawing group.^9,29−32^ In addition to designing new dyes, new hole^33,34^ and electron^35,36^ transport materials were developed to show low glass transition temperatures, low melting points, and high solubility, leading to an increase in pore filling into the semiconductor film and increasing the surface area for the adsorption of the dye. At the end, the semiconductor layer with high dye loading as well as high light-harvesting efficiency capability will ensure the increase in the photocurrent density and the improved performance of DSSCs.^37,38^ Hence, structural/chemical/electronic modifications in any of the components (like D, π, A, linker, interface, etc.) and the resulting electron transfer kinetics^39^ change the PCE significantly.

However, understanding the interplay of different physiochemical and photochemical aspects of the main components of DSSCs demands a molecular level understanding of the dye molecules. This can be achieved by using quantum chemistry methods like density functional theory (DFT) and time-dependent density functional theory (TDDFT), which helped researchers find excellent agreement with experimentally reported geometries and electronic properties.^40,41^ DFT studies on a dye sensitizer molecule^42^ reveal their electron-donating and -withdrawing components which play an important role in understanding intramolecular charge transfer (ICT) after photoexcitation. An easier flow of π-electrons from one conjugate part of the molecule to another part requires that the orbitals are extended through the atoms that make the conjugate system. This implies efficient charge delocalization, thereby improving the intramolecular charge transfer. Hence, the electronic structure of different ionized states of the molecules has to be calculated.^43^ Structural changes due to ICT are possible if the molecule is flexible to adapt the changes provided the π-conjugation is extended through the donor to the acceptor. True ICT states reflect change in both electronic structure and molecular geometry and often prominently differ from the ground states in their molecular structure.^44^

ICT can be addressed in many ways, such as by taking the difference in charge density between two states, by twisting of a certain group in the dye, or with fluorescence.^45,46^ Well-known for efficient ICT, fused molecules have been used in multiple recent studies for designing organic dyes including fused-ring (hereafter referred as fused) molecular structures as D and π-spacers to increase the performance of the dye.^47−49^ Fully fused molecules have planar backbones in all the electronic states, while the nonfused molecular framework usually allows structural distortion. Consequently, the fused ones tend to show higher reorganization energies, resulting in reduced voltage and current losses.^50^ Thus, it is also important to identify the fused molecular groups in a dye molecule and correlate with its reorganization energy and extent of charge transfer.

An understanding of ICT in dye molecules (rather molecules in general) and its correlation to PCE is often captured through expensive simulations and resource-consuming experiments.^51,52^ Usually they involve molecular engineering, screening, strategies for suitable fabrication, and optimization for higher efficiency. During the process often issues like purification, tailoring the absorption band of the sensitizer dye, and cost effectiveness are involved. The question remains, from the huge number of available molecular dyes, how to screen out the ones that show a stronger tendency for high PCE?

In this report, based on DFT and TDDFT methods, we highlight the structural changes and resulting ICT in a dye molecule after photoexcitation (Dye → Dye*/Dye^+^), as shown in Figure 1(II and III). We choose six different dyes with different reported PCE (in parentheses) for comparison (shown in Figure 1(II)(a–f)): ADEKA1 (12.8*%*),^53,54^ C275 (12.5*%*),^55^ C257 (8.7*%*),^56^ D5L2A1 (6*%*),^46^ CB4 (4.28*%*),^57^ and D5L2A3 (2*%*).^46^ While their structures are different, there are ambivalent opinions about a clear distinction of D, π, and A parts in dyes like ADEKA-1 or C275 (as in Figure 1(II)). In C275, the benzothiadizole can be considered as an additional acceptor, and the phenyl between benzothiadizole and −COOH may represent the π-spacer linkage, thereby bringing C275 within the well-established D–A−π–A model.^58^ In the dye ADEKA1, the tetra-thiophene can be chosen as the π-spacer linkage rather than the acceptor. Herein we adopted colored representations based on the mentioned references. These dyes have been studied experimentally and are physically and chemically stable. Here it is worth noting that the performance of the dye molecules, for example ADEKA-1, can be prominently affected by multiple factors, such as the dye synthesis route, choice of electrolytes of different redox potentials, impact of coadsorbents in DSSCs,^59^ assembly of molecular and atomic passivation, electrode–electrolyte interface structures,^60^ cosensitization,^61^ etc. However, our choices of the six dye molecules to understand their structure-dependent ICT and extent of charge transfer over the molecular skeleton still hold valid.

To observe the true ICT in these dyes and to correlate it with the experimentally reported PCE, we use the following steps: (i) The optimized ground-state geometry is used to simulate the UV–vis spectra. (ii) The charge density difference for each transition state is observed. (iii) Then, we select the strongest excited state with the lowest energy to relax the molecule in the excited state. (iv) Next, an electron is removed from the system (to obtain Dye^+^, the cationic state), and we relax the molecular geometry in that cationic state. (v) Similarly, one electron is added to the neutral geometry (to obtain Dye^–^), and the molecule in this anionic state is geometrically relaxed. (vi) Using the final energies of the dye molecule in its relaxed geometries in the ground, cationic, and anionic states, the reorganization energy (λ_i_, i = e, h) is calculated. (vii) The geometrical parameters of the dye in the ground, excited, and cationic states are studied to identify the charge transfer and geometry modification.

As elaborated in a later section, the analysis of the relaxed geometries in the ground, excited, and cationic states of a dye molecule guides us to identify the spatial charge redistribution by means of bond reorganization in different parts of the dye molecule. Note that the consideration of the cationic state helps to mimic the electron injection mechanism from the excited dye molecules into the wide band gap of a semiconductor (like TiO_2_) as usual in experiments.^15^ Finally, we correlate the efficiency of the dye to transfer the electron from the donor to the acceptor with the extent of molecular π-conjugation and structural distortion and charge localization. This should assist in the suitable structural engineering of organic dyes for a more efficient photovoltaic performance.

## Theoretical Methods and Computational Details

The calculations based on the description of the electronic ground state are given by solving the time-dependent Schrödinger equation given by eq 1 for which the Hamiltonian is described by eq 2
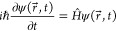
1
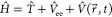
2with kinetic energy *T̂*, electron–electron repulsion *V̂*_ee_, and external potential *V̂*(*r⃗*, *t*). The probability of finding an electron at position *r* at time *t* is given by taking the absolute square of the wave function ψ(*r*, *t*). Based on the Runge–Gross theorem, using the wave function, we can calculate the external potential which will produce the density for further analysis.^62^ Simulations for the dye sensitizer under this study were performed using the ORCA 4.0^63,64^ quantum chemistry package. The ground-state molecular geometries of the dyes and their electronic structures were obtained with the PBE0/def2-TZVP method.^65−67^ The electronic spectroscopy of the dyes and the excited-state geometry optimization were performed using TD-DFT. The PBE0 functional provides a good description of both the ground and excited states,^68^ whereas with the def2-TZVP basis set errors in bond length are smaller than 1 pm and in bond angles smaller than 1°.^66,69^ To reduce the computational time, the RIJCOSX approximation is used in addition to replacing bulky alkyl groups in the dye with smaller alkyl groups. The large hexyl groups in ADEKA1 are replaced by ethyl groups; hexyl groups in C275 and C257 are replaced by methyl groups. Photoabsorption spectra were calculated with the TDDFT approach including a solvent effect to simulate the real experimental conditions, using a conductor like the polarizable continuum model (CPCM).^70^ We calculate the energy gap (*E*_g_) for a dye which is given as the ground-state energy difference between the highest occupied molecular orbital (HOMO) and the lowest unoccupied molecular orbital (LUMO) levels (*E*_g_ = *E*_LUMO_ – *E*_HOMO_). A change in the dipole moment (Δ*S* = *S*_1_ – *S*_0_) of the molecule upon photoexcitation marks charge separation in the molecule. The singlet excitation-state lifetime (τ) is expressed as ,^71^ where *E* is the excitation energy and *f* the oscillator strength; it provides an estimate of the time for the injection of electron from the dye to the semiconductor substrate. The longer the excitation lifetime, the higher the optical stability of the dye in that state.

A semiclassical theory of charge transfer rate equation has been developed by Marcus^72−74^ and quantum mechanically is given by Jortner et al.^75−77^ The electron transfer in the nonadiabatic regime is considered to be analogous to an optical transition between two electronic states within the Franck–Condon approximation.^78^ The rate of charge transfer is the probability of the electron transfer from the donor to the acceptor per second. In the condition of maximum transfer rate, we can consider that the Gibbs free energy is approximately equal to the reorganization energy (λ). The total reorganization energy (λ) is given by λ = λ_e_ + λ_h_, where λ_*i*_ = ( – *E*_±_) + ( – *E*_0_). *E*_0_ and *E*_±_ represent the energies of the neutral and cation or anion species in their lowest energy geometries, respectively, while  and  represent the energies of the neutral and cation species with the geometries of the cation or anion and neutral species.

## Results and Discussion

The ground-state geometry and electronic structures of all six dye molecules as predicted by our calculations show that the energy gap for the dyes lies in the narrow range of 2.5–3.1 eV showing a reasonable match with the earlier reported results (see Supporting Information Table S1). Hence, the choice of functional and basis set are suitable for the present study. The energy of the HOMO of all dyes is lower than the redox potential of the electrolyte (for *I*^–^/*I*^–3^ it is −4.8 eV),^79^ ascertaining that the regeneration of the oxidized dyes is energetically favorable. We also find the LUMO of each chosen dye molecule to be above the conduction band edge position of the semiconductor substrate (for the TiO_2_ semiconductor it is −4.0 eV)^80^ (see Table S1) which guarantees efficient electron injection from the LUMO of the dye molecules to the conduction band of TiO_2_.

**Figure 2:**
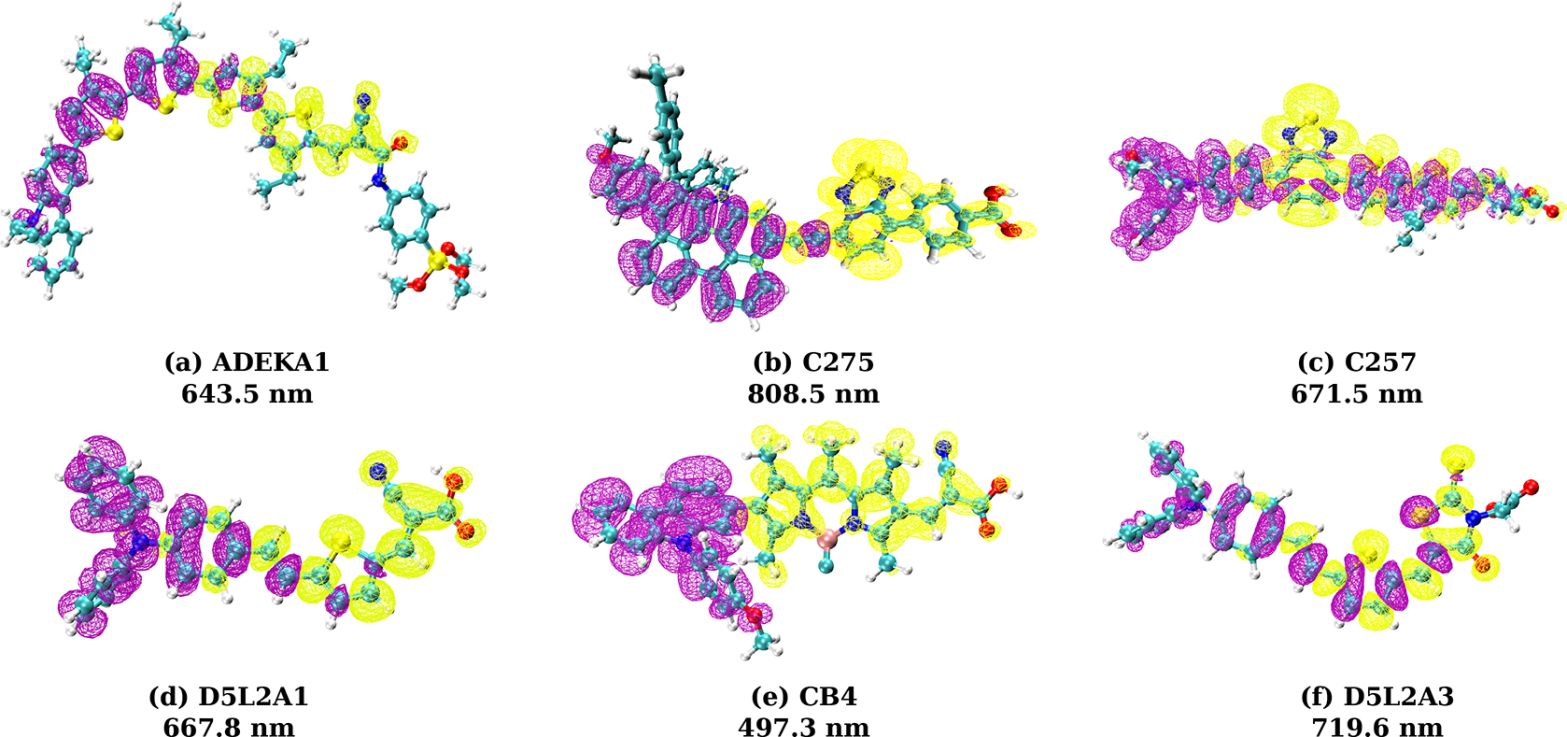
Localization of hole and electron density on each dye in its excited state. (a) ADEKA1 shows the hole density (yellow color isosurface) on carbazole (donor) and the first two thiophene and the electron density (purple color isosurface) on cyanide and two thiophene groups (acceptor). (b) C275 shows the hole density on indenoperylene (donor) and electron density on ethynyl benzothia-diazole-benzoic acid (acceptor). (c) C257 shows the hole density localization on triphenyl amine (donor) and the electron density localized on the benzo-dithiazole group (acceptor). (d) CB4 shows the hole density on carbazole (donor) and the electron density on cyanoacetic acid (acceptor). (e) D5L2A1 has the hole density on triphenyl amine (donor) and the electron density on cyanoacrylate (acceptor). (f) D5L2A3 shows the hole density on triphenyl amine (donor) and the electron density on rhodanine (acceptor). Atoms (color): C (cyan), N (blue), O (red), S (yellow), H (gray), B (pink), and Si (dark yellow).

Using TD-DFT and CPCM combined calculations, photoabsorption excited states were obtained with the solvent effect as given in experimental studies (see Figure S1). For example, toluene as a solvent for ADEKA1,^53,54^ tetrahydrofuran (THF) solvent for C257^56^ and C275,^55^ methyl chloride (CHCl_3_) solvent for CB4,^57^ and methyl cyanide (MeCN) solvent for D5L2A1^46^ and D5L2A3^46^ have been used in our calculations (following given experimental references). The oscillator strength and excited-state lifetime (τ) are tabulated in Supporting Information Table S2. The photoabsorption spectra reveal that the first strong excitation is an outcome of the transition from the ground to first excited state in the case of ADEKA1 and C275, to the second excited state for D5L2A1 and D5L2A3, third excited state for C257, and sixth excited state for CB4. The contributions of molecular orbital transitions in a given excited state are tabulated in Table S2. From Table S2 we observe that ADEKA1 (at 643.5 nm), C275 (at 808.5 nm), C257 (at 671.5 nm), and D5L2A3 (at 719.6 nm) show 90*%* HOMO → LUMO transition. D5L2A1 (at 667.8 nm) shows 97*%* HOMO–1 → LUMO+1 transition, and CB4 (at 497.3 nm) shows 25*%* HOMO → LUMO transition for the first strong excitation. Hence, it is worth noting that the first strong excitation does not necessarily originate mainly from the HOMO → LUMO transition. In Figure 2a–f we plot the charge density difference (with respect to the ground state) for the first strong excited states (as reported above) and observe that all six dyes show charge migration from the donor group to the acceptor group. To study how the photoexcitation-induced ICT and the modified molecular geometry of the dye molecule affect each other, we will optimize the excited-state geometry of dyes in their first strong excited state (we will refer to the “first strong excited state” as the “excited state” throughout this article).

A comparison of the dipole moment between the ground-state and the excited-state geometries of dyes is given in Supporting Information Table S1. The dipole value for ADEKA1, C275, and D5L2A3 increases upon transition from the ground to excited state, suggesting possible prominent modification of charge densities and molecular structural parameters.^81,82^ On the contrary in the case of C257 and D5L2A1, a decrease in the dipole value is found, which could be due to electron density localized on a group other than the acceptor^83^ after photoexcitation (see Figure 2). In the case of CB4 the geometry optimization calculations for the excited state did not reach convergence, so we keep CB4 out in our further discussions. Figure 2c shows that the spatial localization of the electron density in the excited state is mainly on the benzothiadiazole unit, which is far away from the substrate when compared to its ground-state charge distribution. The relatively small excited-state dipole moment in C257 (*S*_*n*_ = 3.604 D, as enlisted in Table S1, corresponding to the excitation with 671.5 nm) indicates a small excited-state charge separation. This suggest that excited-state charge density localization could be used as one important and cheaper way to understand charge separation in the excited state. This could also explain the decrease in the dipole moment upon excitation of C257 with a 671.5 nm photon.

The increase in the dipole moment in the excited-state geometry shows prominent charge separations,^83^ as also supported from our charge density distribution results for C275 (see Figure 2b). Electron and hole reorganization energies (λ_*i*_, with *i* = e for electron, h for hole) are other important parameters, showing the efficiency of the dye to transport holes or electrons across the molecule. Note that a smaller difference in the electron and hole reorganization energy reveals the efficiency of the dye in transporting both charged particles with similar ease. In addition, analysis of the λ_*i*_ helps in the choice of the charge transport material.^84^Table 1 shows (λ_h_ < λ_e_) for D5L2A1, CB4, and D5L2A3, which show good hole transfer ability, whereas (λ_h_ > λ_e_) for C257 shows better electron transfer ability. ADEKA1 and C275 show a small magnitude of λ′, i.e., a small difference between λ_h_ and λ_e_, suggesting a similar efficiency of these molecules in electron and hole transfer abilities (in comparison to other dyes). The presence of fused groups is known to affect ICT and reorganization energies owing to less distorted structures. However, they are also known to sometimes hinder the ICT process.^85^ To address the effect of the presence and absence of fused groups at the donor and π-spacer sites (see Table 1), we analyze the structural changes in the different parts of the dye molecules followed by the photoexcitation and removal of electrons simultaneously identifying the ICT pathway and extent of π-conjugation.

**Table 1:** Presence of Fused D, π-Spacer, and A Group in the Dye, Calculated Electron (λ_e_) and Hole (λ_h_) Reorganization Energy, Total Reorganization Energy (λ), and Reorganization Energy Difference (λ′) between λ_e_ and λ_h_ for ADEKA1, C275, C257, CB4, D5L2A1, and D5L2A3^a^

dyes	fused D	fused π	fused A	λ_e_	λ_h_	λ	λ′ = |λ_e_ – λ_h_|
ADEKA1	yes	NA	no	0.4507	0.4441	0.8949	0.0066
C275	yes	NA	no	0.4077	0.4509	0.8587	0.0432
C257	no	NA	no	0.0416	0.2483	0.2900	0.2067
D5L2A1	no	no	no	0.4063	0.1808	0.5817	0.2255
CB4	yes	yes	no	0.3384	0.3014	0.6399	0.0370
D5L2A3	no	no	no	0.3523	0.2908	0.6431	0.0615

a NA means not applicable.

**Figure 3:**
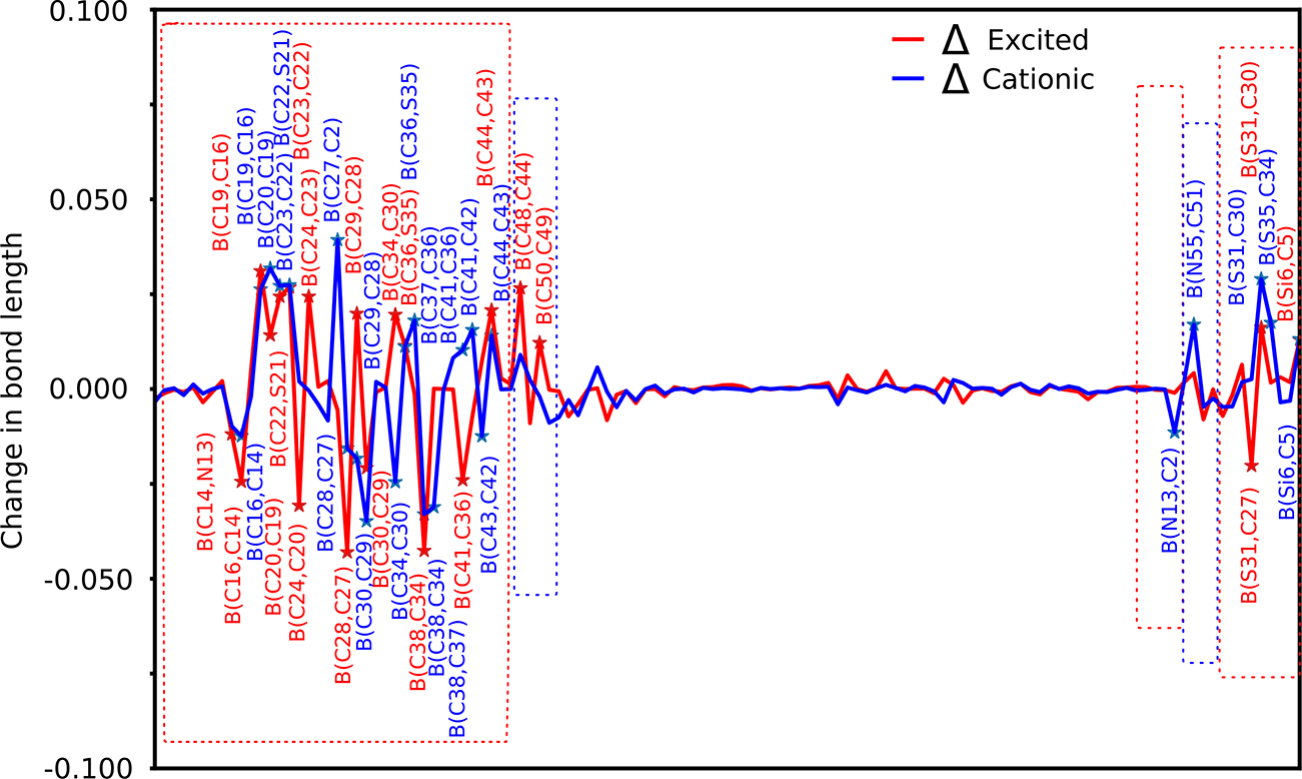
Difference in bond length of the ADEKA1 dye in the excited (red) and cationic states (blue) with respect to the ground-state geometry. The difference (Δ > 0.01 Å) is marked with “*”. The red box highlights changes for the acceptor group, and the blue box highlights changes for the donor group.

**Figure 4:**
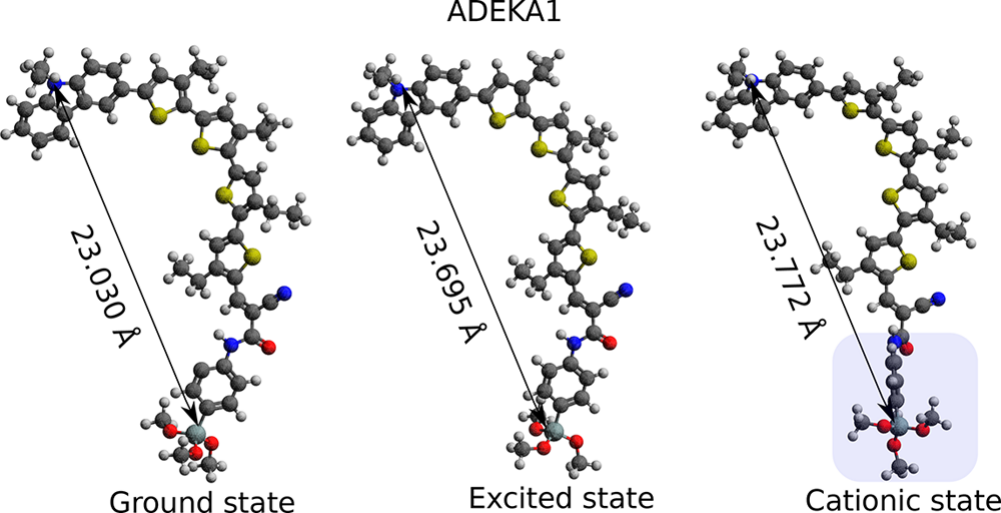
Change in the distance between the donor and acceptor group of ADEKA1 in the ground state, excited state, and cationic state. Upon removal of the electron (cationic state), ADEKA1 shows a highly planar acceptor group (blue highlighted box).

In Figure 3 we show the difference in bond length between the ground-state and excited-state geometry (red plot) and ground-state and cationic-state geometry (blue plot). Corresponding numbers for these bond length differences are listed in Supporting Information Table S3, columns Δ*E* and Δ*C*. The abbreviation for bond length between the atom type X and its index *n* (X*n*) is given by B(X*i*, X*j*), angle as A(X*i*, X*j*, X*l*), and dihedral angle as D(X*i*, X*j*, X*l*, X*k*). The atomic indices for atoms in ADEKA1 except hydrogen in donor groups are from 48 to 62 and for acceptor from 0 to 47. The variations in bond length between B(C48, C44) and B(C50, C49) of the donor group and B(C44, C43), B(C36, S35), B(C22, S21), B(C36, S35), B(S31, C30), and B(Si6, C5) in the acceptor group show the extended conjugation in the ADEKA1 dye. Furthermore, a change in angles and dihedral angles in acceptor groups demonstrates the delocalization of charge throughout the structure of ADEKA1 (see Figure S4a for bonds angles, Figure S5a for dihedral angles, and Table S3 for corresponding values). The overall effect of extended π-conjugation and delocalization of charge, as observed in the molecular frame of the dye in excited and cationic states with respect to the ground state (see Figure 3 and Figure 4), is summarized as follows. The alteration of the structural parameters for the entire molecule describes that the π-conjugation is extended in both excited and cationic states. The distance between the nitrogen (blue atom) in the donor group and the silicon atom in the acceptor group increases as the structure evolves from ground (23.030 Å) → excited state (23.695 Å) → cationic state (23.772 Å). Finally, we estimate the twist from the dihedral angle between the donor and the first thiophene of the acceptor, and also between the last thiophene and the phenyl-silyl-anchor (blue shaded box in Figure 4 for the cationic state). ADEKA1 has a fused donor group (see Table 1), and prominent variation is observed at the atomic sites of the donor in the close vicinity of the oligothiophene in the acceptor group (B(C48, C44), B(C50, C49), D(C49, C48, C44, C43), and D(C49, C48, C44, S45)). This shows that the fused rings are structurally stable upon excitation and ionization. The oligothiophene, not being fused, shows variation along the complete structure and reflects the transfer of charge from one end of the dye to another. The distinctive twist of the phenyl-silyl-anchor in the cationic state (highlighted box in Figure 4) suggests a possibility for efficient charge transferred from the dye to the semiconductor substrate.^86−88^ Similarly, we study the optimized geometry of all the other molecules in the study in Supporting Information Figures S3, S4, and S5. The atomic indices (excluding hydrogen) for donor and acceptor parts of C275 are as follows: (i) in the donor from 0 to 41 and (ii) 42 to 63. C275 shows variation in the bond lengths along B(C6, C5), B(C14, C12), B(C38, C32), and B(O28, C9) of the donor and B(C44, C37), B(C50, C49), B(C60, C55), and B(C61, C57) of the acceptor (see Figure S3a for bond lengths and corresponding values in Table S4). However, major variations in the bond angles and dihedral angles are seen on the donor group (see Figure S4b for bonds angles, Figure S5b for dihedral angles, and Table S4). This is reflected in Figure S6 (second column for C275) where the cationic-state geometry shows the twist in the donor along the link between the donor and acceptor (see corresponding dihedral angle D(C50, C46, C37, C36)) when in the excited state. In the cationic state a twist by 24.48° (D(C51, C46, C37, C33)) between the donor and acceptor and by 21.76° (D(C59, C55, C48, C47)) in the acceptor is observed, resulting in a planar structure (Figure S3b for bond lengths, Figure S4c for bond angles, Figure S5c for dihedral angles, and Table S4). This favors the charge transfer from the donor to acceptor. C275 has the largest fused structure in the donor part; however, a prominent variation in the bond length at B(C6, C5), B(C14, C12), B(C38, C32), and B(O28, C9) in the donor shows a less stable structure. On the other hand, twisting in the acceptor shows the transfer of charge between the rings.

The atomic indices (excluding hydrogen (H)) for C257 in its three parts are as follows: (i) in the donor, carbon (C) from 6 to 45, nitrogen (N) 46, 47, and 29, oxygen (O) 42 and 43, and sulfur (S) 48, 53, and 52; (ii) in the acceptor, C from 0 to 5 and 49 and O 50 and 51. In C257 the donor becomes planar in the cationic state; however, the acceptor also goes out of plane with respect to the donor. This might affect the effective charge transfer to the substrate (Supporting Information Figure S6 (third row in third column for C257), Figure S3b for bond lengths, Figure S4c for bonds angles, Figure S5c for dihedral angles, and corresponding values in Table S5). The variation of structural parameters in the donor at site (B(C7, C6)), the nonfused site between cyclopentadithiophene and benzodithiazole (B(C16, C15)) after excitation, and the nonfused site B(C17, C16) after ionization along with twisting between the donor and acceptor) hints at active charge transfer between the rings. Negligible structural variation inside the cyclopentadithiophene group reveals a stable structure with minimal distortion which results in low electron recombination energy for the C257 dye.

The atomic indices (excluding hydrogen) for D5L2A1 in its three parts are as follows: (i) donor, from 0 to 18, (ii) π-spacer, from 19 to 25, and (iii) acceptor, from 26 to 30. D5L2A1 shows a prominent bond length variation (change (Δ) > 0.02 Å with respect to ground state) on the entire molecule; however, the dihedral angle varies mostly along the donor group (see Supporting Information Figure S3c for bond lengths, Figure S4d for bonds angles, Figure S5d for dihedral angles, and values in Table S6). In this system, electronic charges are mostly localized near the donor, with negligible transfer to the π-spacer and acceptor (Figure S6 (fourth column for D5L2A1)), suggesting a narrow charge conjugation. The atomic indices (excluding hydrogen) for D5L2A3 in its three parts are as follows: (i) in the donor, from 0 to 18, (ii) in the π-spacer, from 19 to 25, and (iii) in the acceptor, from 26 to 37. In D5L2A3 we observe very few variations in structural parameters showing less effects when in excited and cationic states (see Supporting Information Figure S3d for bond lengths, Figure S4e for bonds angles, Figure S5e for dihedral angles, and values in Table S7 and Figure S6 (sixth column for D5L2A3)). The absence of any fused structures in D5L2A1 and D5L2A3 dyes might also contribute to the less efficient charge transfer.

## Summary and Conclusion

The ground-state geometry for the selection of experimentally synthesized dye molecules ADEKA1, C275, C257, D5L2A1, CB4, and D5L2A3 is optimized using DFT. The UV–vis spectra and band gap computed using the ground-state geometry and TDDFT method are found to be a reasonable match with the reported experimental results (see Supporting Information Figure S1). Our charge density difference plots for excited states computed using TDDFT highlight the different response of the dyes. While dyes like ADEKA1, C275, D5L2A1, CB4, and D5L2A3 show effective charge separation and transfer from the donor to acceptor (see Figure 2 for the first strong excited state and Supporting Information Figure S2 for other excitation states), for C257 the charge appears to be localized on the donor instead of the acceptor group. Hence, our results for charge density difference maps capture the charge redistribution for different excitation states at a reasonable computational cost. The estimated electron and hole reorganization energy (λ_i_, i = e, h) suggests that ADEKA1 and C275 possess better electron and hole transfer abilities than other dyes. Additionally, these two dyes show a significant increase in dipole moment (>2 D in going from the ground to excited state), which is consistent with their reported high efficiency.

Further analysis based on the comparison and difference in the structural parameters of the dyes (i) between ground and excited states and (ii) between ground and cationic states guided us to establish a more general correspondence between the molecular structural parameters, calculated electronic properties, and resulting efficiency of the dyes. Additionally, the presence of fused structures as only the donor in ADEKA1 and C275 and as only the π-spacer in CB4 has been observed to play an important role in supporting the understanding of ICT through structural changes. This study also finds that the presence of the fused structure as the donor group results in a smaller difference between the hole and electron recombination energy. The presence of the fused structure as only the π-spacer in D−π–A is observed to lower the electron recombination energy; however, this is not yet conclusive, and further study on this topic could be of interest for the reader. Analyzing the performance of the dye solely based on the recombination energy or the dipole moment is not conclusive, and hence structural analysis of dyes in various excited and ionic states helps one to understand the ICT more effectively. The dyes like ADEKA1 (Figure 4), C275, and C257 (Supporting Information Figure S6) show prominent changes in the structural parameters in the donor and acceptor region, in both excited and cationic states. On the other hand, D5L2A1 demonstrates changes in the π-spacer and in the donor group, respectively. However, no significant structural parameter changes were observed for D5L2A3.

Interestingly, we observe that efficient dyes show extended charge transfer, with a higher possibility of charge migration over the molecular skeleton. The extent of charge transfer is directly related to the electron-donating capacity of the donor, the withdrawal capacity π-spacer and/or acceptor groups, and the extent of conjugation length and associated structural rearrangement of the groups. Our study of detailed structural modifications in photoexcited dye molecules and associated loss of electron, accompanied by the calculation of the resulting electron and hole reorganization energy, reasonably explains why certain dye molecules show high photoconversion efficiency in comparison to others and can assist in improving the design of the dyes for DSSCs. While we selected certain dye molecules as a prototype, our approach proves to be simple yet significant for any dye molecule, to indeed aid the experimentalists to pre-estimate/manipulate the charge transfer process in such molecules, which in general has a critical role in physics, chemistry, biology, and material science.

## Supporting Information Available

Tables and figures for changes in structural parameters for all dyes; additional information regarding UV–vis spectroscopy and electronic transitions (PDF)
