# Supplementary material for: Photoexcited Intramolecular Charge Transfer in Dye Sensitizers: Predictive In Silico Screening for Dye-Sensitized Solar Cell Devices
Source: ACS Omega. 2022 Apr 13;7(16):13465–74. doi: 10.1021/acsomega.1c06233 (PMC9088764; doi:10.1021/acsomega.1c06233)
Supplement: Supplementary file 1 — ao1c06233_si_001.pdf [file ao1c06233_si_001.pdf]

Supplimentary Information
Photo-excited Intramolecular Charge Transfer In Dye Sensitizers : Predictive In-silico Screening for Dye Sensitized Solar Cell Devices

Kalyani Chordiya,[†,‡] Md. Ehesan Ali,[¶] and Mousumi U. Kahaly[*,†,‡]

†*ELI-ALPS, ELI-HU Non-Profit Ltd., Wolfgang Sandner utca 3., Szeged, H-6728, Hungary*

‡*Institute of Physics, University of Szeged, Dóm tér 9, H-6720 Szeged, Hungary*

¶*Institute of Nano Science and Technology, Mohali, Punjab 140306, India*

E-mail: Mousumi.UpadhyayKahaly@eli-alps.hu

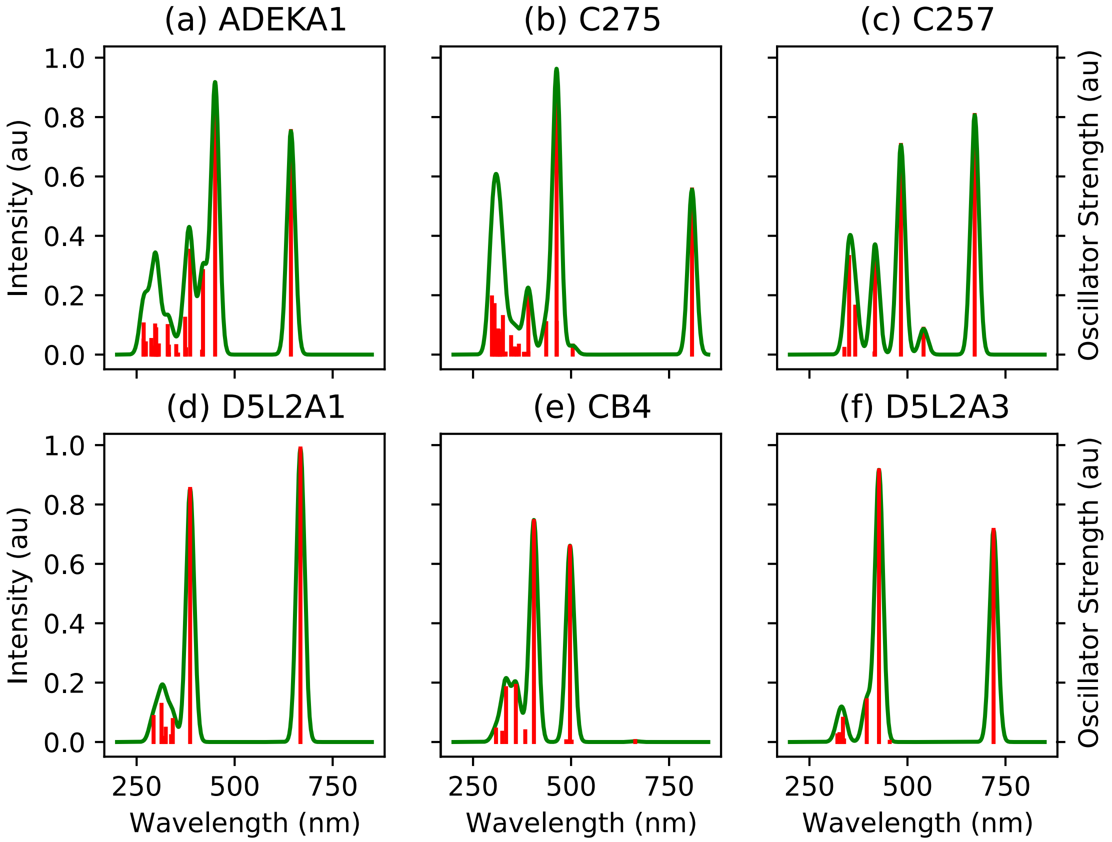

Figure S1: UV-VIS absorption TDDFT spectra calculated for (a) ADEKA1, (b) C275, (c) C257, (d) CB4, (e) D5L2A1 and (f) D5L2A3; is plot with Gaussian broadening of full width half maxima (FWHM) of 10 nm.

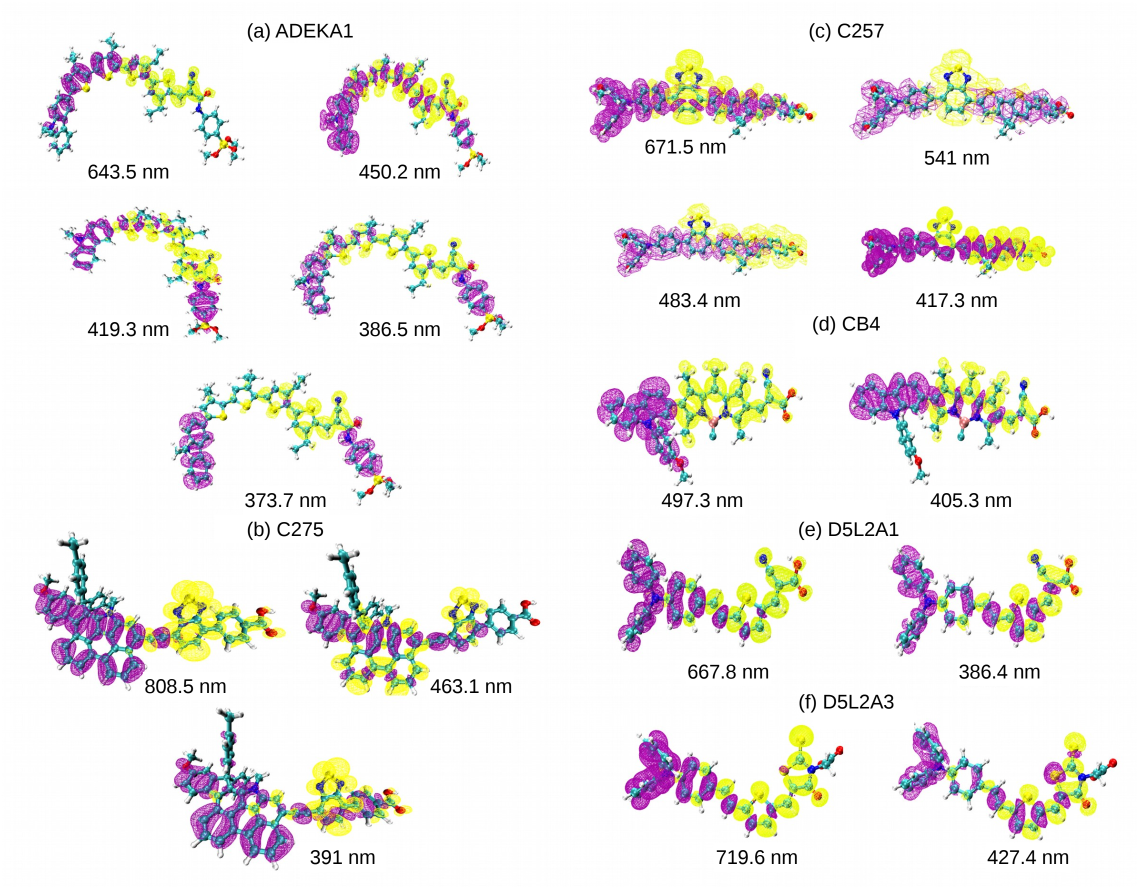

Figure S2: Charge density difference maps for (a) ADEKA1, (b) C275, (c) C257, (d) CB4, (e) D5L2A1 and (f) D5L2A3 in different excited states. The purple colour show decrease in electron density and yellow colour show the increase in electron density. [Atoms (colour): C (cyan), N (blue), O (red), S (yellow), H (gray), B (pink), Si (dark yellow)]

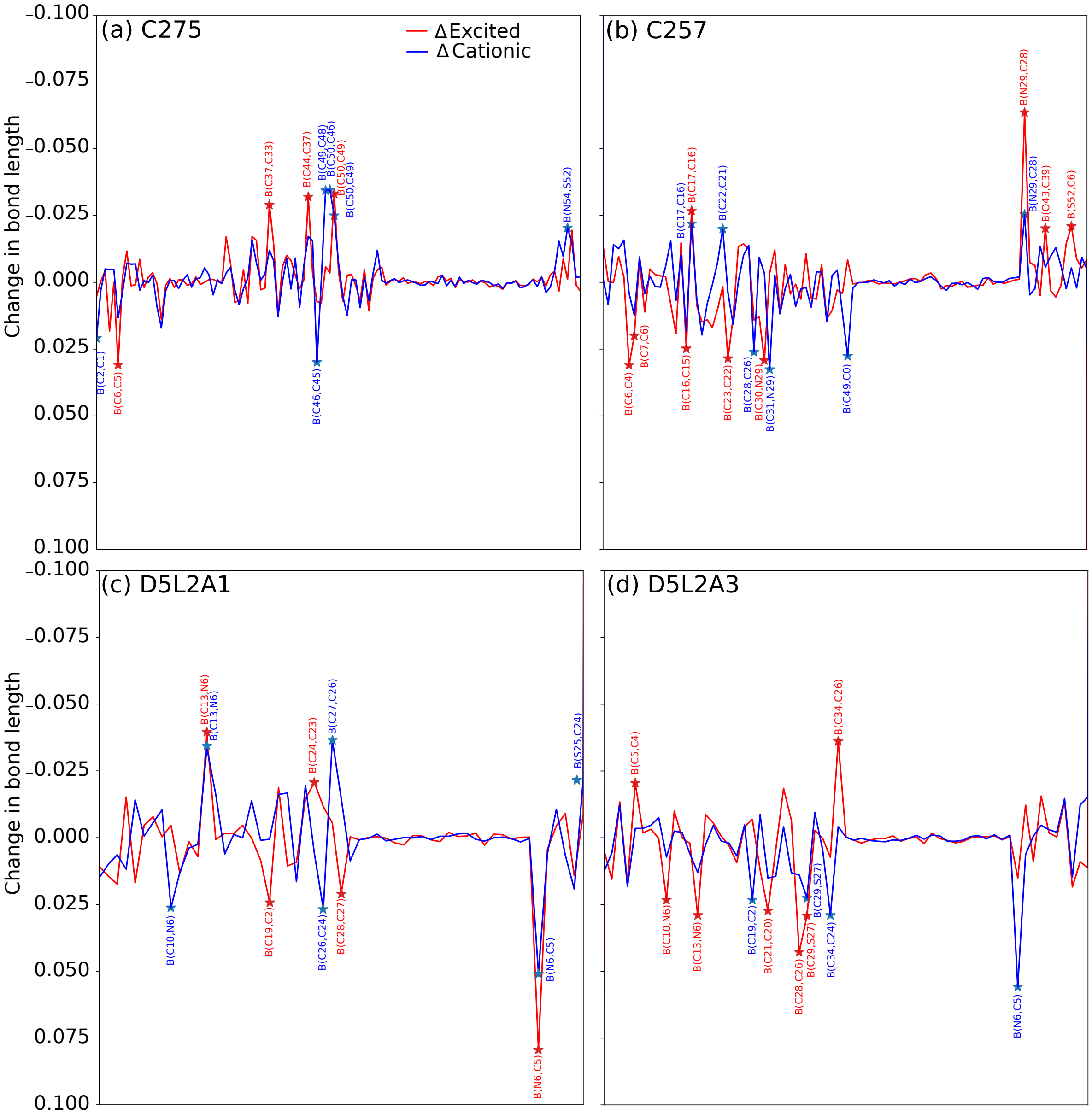

Figure S3: Variations in bond length for (a) C275, (b) C257, (c) D5L2A1 and (d) D5L2A3; between ground state and excited state (red) and ground state and cationic state (blue). *for Δ >0.02Å.

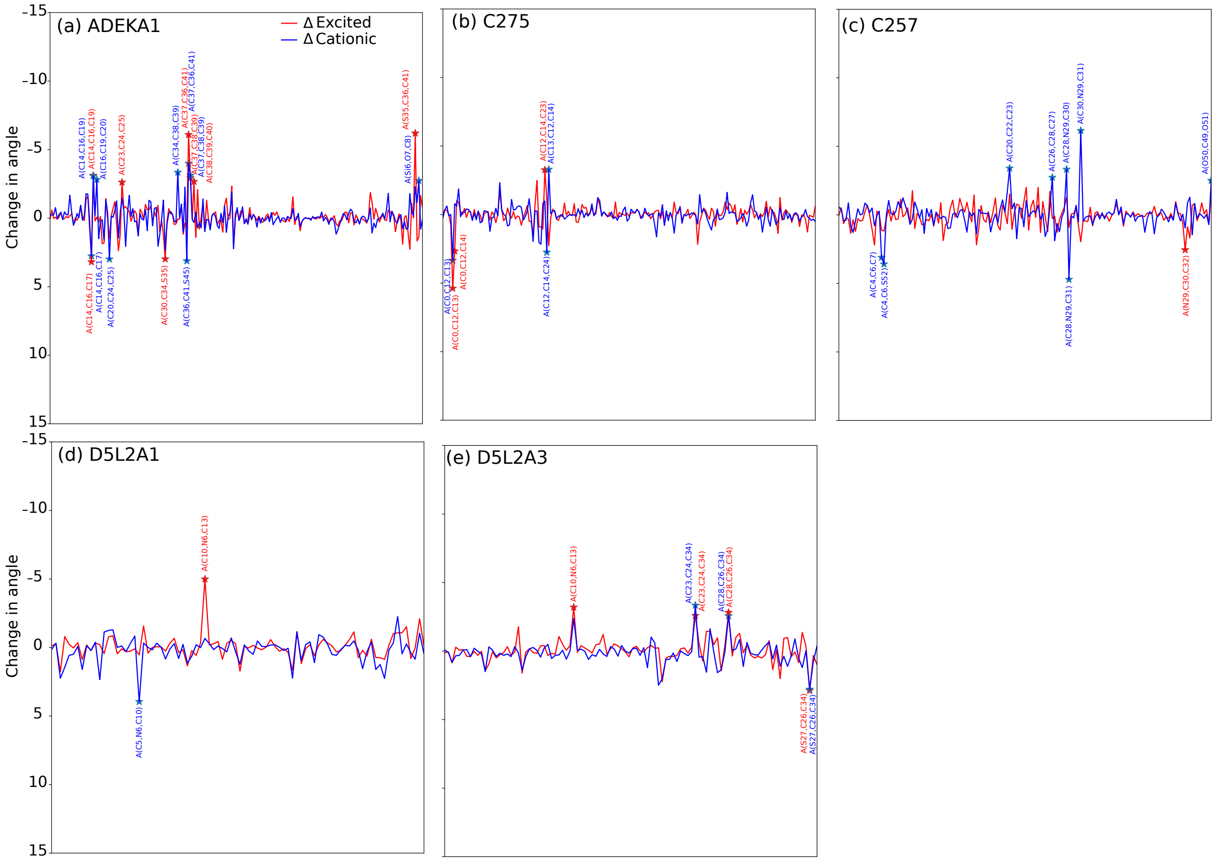

Figure S4: Variations in bond angle for (a) ADEKA1, (b) C275, (c) C257, (d) D5L2A1 and (e) D5L2A3; between ground state and excited state (red) and ground state and cationic state (blue). *for $\Delta > 2°$.

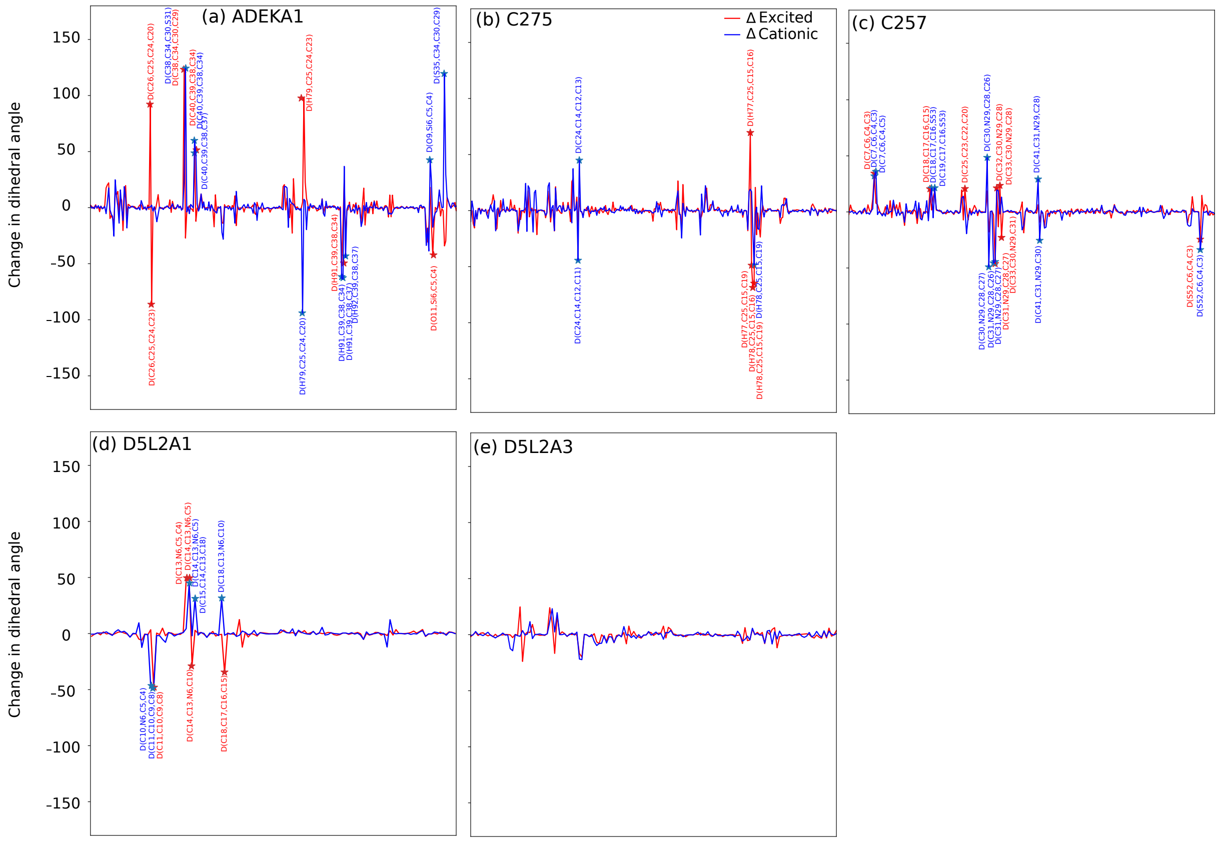

Figure S5: Variations in dihedral angle for (a) ADEKA1, (b) C275, (c) C257, (d) D5L2A1 and (e) D5L2A3; between ground state and excited state (red) and ground state and cationic state (blue). *for $\Delta > 40°$.

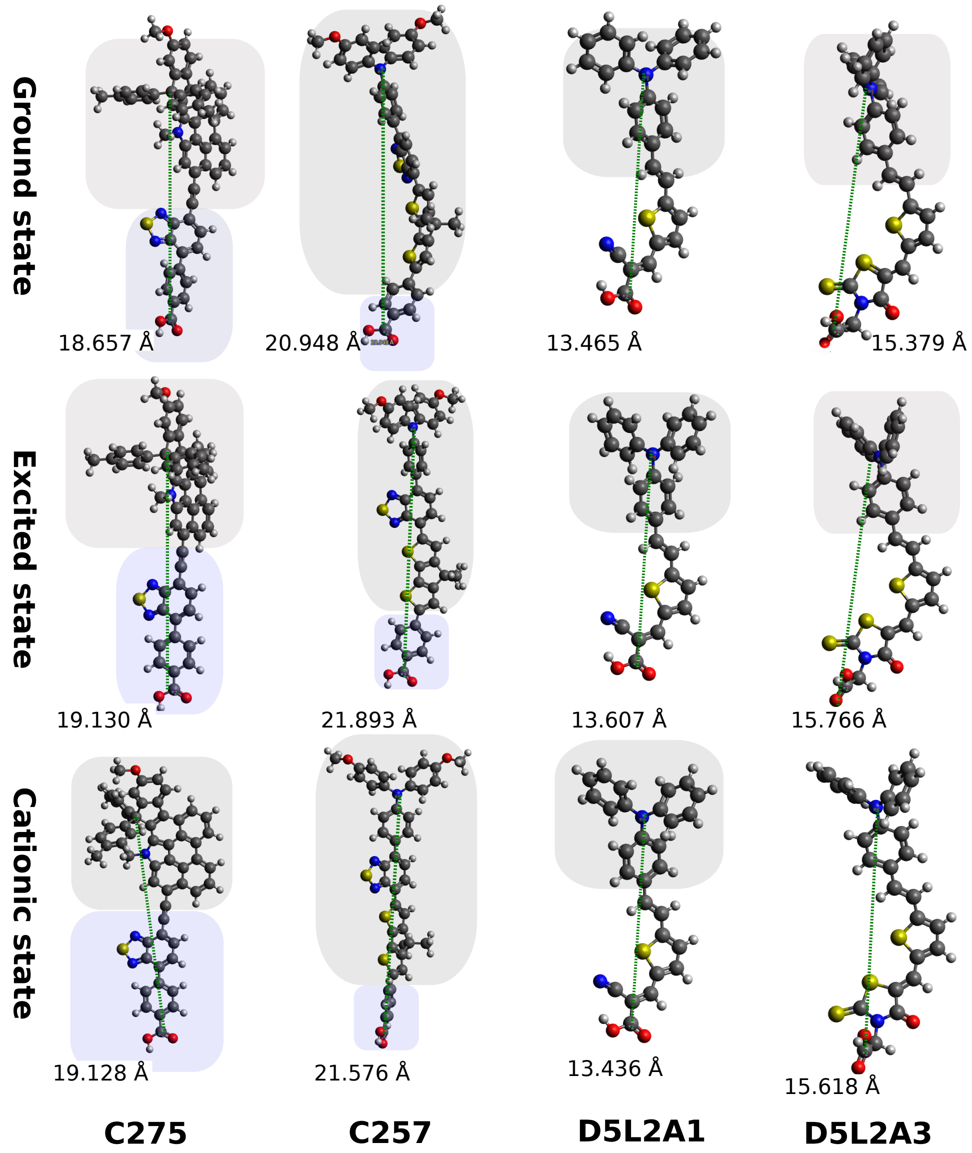

Figure S6: Change in the distance between the donor and acceptor group (green dotted line) of dye molecules in ground state, excited state and cationic state. Gray box for donor, blue box for acceptor highlights the planar behaviour in dye when in excited and cationic state.

Table S1: Energy of HOMO, LUMO, Energy gaps (*reference value), ground state dipole moment (S_0) and ground state dipole moment in excited state (S_n). For CB4 geometry optimization did not converge for sixth excited state.

Species	E_{HOMO}(eV)	E_{LUMO}(eV)	E_g(eV) (reference)	(S_0)(debye)	(S_n)(debye)
**ADEKA1**	-5.41	-2.68	2.73 (*1.85[1])	12.24	(S_1) 14.26
**C275**	-5.20	-2.80	2.40 (*2.07[2])	5.76	(S_1) 7.91
**C257**	-5.18	-2.59	2.59 (*1.86[3])	6.43	(S_3) 3.60
**D5L2A1**	-5.48	-2.93	2.55 (*2.42[4])	10.32	(S_2) 7.72
**CB4**	-5.99	-2.88	3.11 (*1.92[5])	6.39	–
**D5L2A3**	-5.46	-2.94	2.52 (*2.18[4])	7.62	(S_2) 8.27

Table S2: Calculated the excitation lifetime τ (ns), molecular orbital contribution for the excitation states with high oscillator (OS) strength for ADEKA1, C275, C257, CB4, D5L2A1 and D5L2A3.

Dye	λ in nm (Excited state)	Molecular orbital contribution	OS	$\tau(ns)$
**ADEKA1**	643.5 (1)	$HOMO \rightarrow LUMO(97\%)$	0.75	8.27
	450.2 (2)	$HOMO-1 \rightarrow LUMO(84.39\%)$	0.91	3.33
		$HOMO \rightarrow LUMO+1(11.10\%)$		
	419 (3)	$HOMO-2 \rightarrow LUMO(47.79\%)$	0.28	9.41
		$HOMO \rightarrow LUMO+2(36.37\%)$		
	386.5 (4)	$HOMO \rightarrow LUMO+1(37.36\%)$	0.35	6.39
		$HOMO-4 \rightarrow LUMO(29.42\%)$		
		$HOMO-2 \rightarrow LUMO(26.41\%)$		
	373.7 (6)	$HOMO-3 \rightarrow LUMO(44.57\%)$	0.12	17.4
		$HOMO-4 \rightarrow LUMO(38.13\%)$		
**C275**	808.5 (1)	$HOMO \rightarrow LUMO(97.87\%)$	0.556	17.619
	463.1 (2)	$HOMO \rightarrow LUMO+1(91.32\%)$	0.851	3.77
	391 (6)	$HOMO-3 \rightarrow LUMO(82.42\%)$	0.21	10.61
**C257**	671.5 (3)	$HOMO \rightarrow LUMO(97.05\%)$	0.8	8.37
	541 (5)	$HOMO-1 \rightarrow LUMO(92.42\%)$	0.08	50.4
	483.4 (6)	$HOMO \rightarrow LUMO+1(90.46\%)$	0.70	4.96
**D5L2A1**	667.8 (2)	$HOMO-1 \rightarrow LUMO+1(96.96\%)$	0.99	6.76
	386.4 (5)	$HOMO-1 \rightarrow LUMO(41.22\%)$	0.85	2.62
		$HOMO \rightarrow LUMO+1(15.36\%)$		
**CB4**	497.3 (6)	$HOMO-1 \rightarrow LUMO(54.28\%)$	0.65	5.62
		$HOMO \rightarrow LUMO(25.78\%)$		
		$HOMO-2 \rightarrow LUMO(14.22\%)$		
	405.3 (8)	$HOMO-2 \rightarrow LUMO(77.10\%)$	0.74	3.31
**D5L2A3**	719.6 (2)	$HOMO \rightarrow LUMO(97.19\%)$	0.71	10.8
	427.4 (6)	$HOMO-1 \rightarrow LUMO(89.43\%)$	0.91	2.99

Table S3: Changes of bond length (B) in angstroms, bond angle (A) and dihedral angle (D) in degrees for ADEKA1 during relaxation from ground state to excited state (ΔE) and cationic state (ΔC). Change in bond length, bond angle or dihedral angle connecting donor and acceptor is highlighted by green box.

Definition	ΔE	Definition	ΔC
B(C14,N13)	-0.0119	B(C16,C14)	-0.0124
B(C16,C14)	-0.0244	B(C19,C16)	0.0263
B(C19,C16)	0.0311	B(C20,C19)	0.0318
B(C20,C19)	0.0142	B(C22,S21)	0.0272
B(C22,S21)	0.0244	B(C23,C22)	0.0274
B(C23,C22)	0.0268	B(C27,C22)	0.0393
B(C24,C20)	-0.0307	B(C28,C27)	-0.0156
B(C24,C23)	0.0244	B(C29,C28)	-0.0183
B(C28,C27)	-0.043	B(C30,C29)	-0.0348
B(C29,C28)	0.0199	B(C34,C30)	-0.0245
B(C30,C29)	-0.0208	B(C36,S35)	0.0112
B(C34,C30)	0.0196	B(C37,C36)	0.0181
B(C36,S35)	0.0114	B(C38,C34)	-0.033
B(C38,C34)	-0.0426	B(C38,C37)	-0.0311
B(C41,C36)	-0.024	B(C41,C36)	0.0103
B(C44,C43)	0.0208	B(C42,C41)	0.0156
B(C48,C44)	0.0266	B(C43,C42)	-0.0124
B(C50,C49)	0.0122	B(C44,C43)	0.0141
B(S31,C27)	-0.0202	B(N13,C2)	-0.0114
B(S31,C30)	0.0162	B(N55,C51)	0.017
B(Si6,C5)	0.013	B(S31,C30)	0.029
A(C14,C16,C17)	3.19	A(C14,C16,C17)	2.76
A(C14,C16,C19)	-3.12	A(C14,C16,C19)	-3.08
A(C23,C24,C25)	-2.62	A(C16,C19,C20)	-2.82
A(C30,C34,S35)	2.98	A(C20,C24,C25)	2.99
A(C37,C36,C41)	-6.09	A(C34,C38,C39)	-3.34
A(C37,C38,C39)	-2.96	A(C36,C41,S45)	3.12
A(C38,C39,C40)	-2.67	A(C37,C36,C41)	-4
A(S35,C36,C41)	-6.2	A(C37,C38,C39)	-3.12
		A(Si6,O7,C8)	-2.74
D(C8,O7,Si6,O9)	20.34	D(C12,O11,Si6,C5)	-25.41
D(C8,O7,Si6,O11)	22.14	D(C12,O11,Si6,O7)	24.85
D(C17,C16,C14,N13)	-20.39	D(C38,C34,C30,C29)	124.34
D(C26,C25,C24,C20)	92.22	D(C38,C34,C30,S31)	59.92
D(C26,C25,C24,C23)	-86.23	D(C40,C39,C38,C34)	48.74
D(C38,C34,C30,C29)	123.29	D(C40,C39,C38,C37)	-21.99
D(C38,C34,C30,S31)	-27.14	D(C42,C41,C36,C37)	-28.17
D(C40,C39,C38,C34)	50.45	D(C49,C48,C44,C43)	-93.96

Definition	ΔE	Definition	ΔC
D(C42,C41,C36,C37)	-12.28	D(C49,C48,C44,S45)	-62.86
		D(H79,C25,C24,C20)	-63.83
D(H68,C8,O7,Si6)	20.54	D(H91,C39,C38,C34)	36.82
D(H74,C12,O11,Si6)	-22.71	D(H91,C39,C38,C37)	-44.39
D(H75,C12,O11,Si6)	25.86	D(H92,C39,C38,C34)	-33.69
D(H79,C25,C24,C23)	97.68	D(H92,C39,C38,C37)	-38.65
D(H80,C25,C24,C20)	22.72	D(O7,Si6,C5,C0)	42.56
D(H91,C39,C38,C34)	-50.73	D(O9,Si6,C5,C0)	23.07
D(H92,C39,C38,C34)	24.38	D(O9,Si6,C5,C4)	119.53
D(H92,C39,C38,C37)	-29.29	D(O11,Si6,C5,C0)	-25.23
D(O7,Si6,C5,C4)	-30.69	D(S35,C34,C30,C29)	-12.19
D(O11,Si6,C5,C4)	-42.21	D(S35,C34,C30,S31)	29.65

Table S4: Changes of bond length in angstroms, angle and dihedral angle in degrees for C275 during relaxation from ground state to excited state (ΔE) and cationic state (ΔC). Change in bond length, bond angle or dihedral angle connecting donor and acceptor is highlighted by green box.

Definition	ΔE	Definition	ΔC
B(C5,C0)	0.0183	B(C2,C1)	0.0209
B(C6,C5)	0.0309	B(C6,C5)	0.0132
B(C8,C7)	-0.0117	B(C14,C12)	0.0171
B(C14,C12)	0.0138	B(C34,C27)	-0.0159
B(C27,C2)	-0.017	B(C37,C33)	-0.012
B(C34,C27)	-0.0172	B(C38,C32)	0.0129
B(C34,C33)	-0.0157	B(C44,C37)	-0.0172
B(C37,C33)	-0.029	B(C45,C44)	-0.0155
B(C37,C36)	-0.0141	B(C46,C45)	0.0299
B(C38,C32)	0.0123	B(C49,C48)	-0.0344
B(C39,C38)	-0.0101	B(C50,C46)	-0.0347
B(C44,C37)	-0.032	B(C50,C49)	-0.025
B(C50,C49)	-0.0332	B(C55,C48)	0.0123
B(C60,C55)	0.0106	B(C61,C57)	-0.012
B(O28,C9)	-0.0195	B(N53,S52)	-0.0154
nan	NaN	B(N54,S52)	-0.0204
nan	NaN	B(O28,C9)	-0.015
A(C0,C12,C13)	5.38	A(C0,C12,C13)	3.34
A(C0,C12,C14)	2.66	A(C12,C14,C24)	2.75
A(C12,C14,C23)	-3.26	A(C13,C12,C14)	-3.3
D(C14,C12,C11,C10)	-21.43	D(C13,C12,C11,C6)	22.16
D(C18,C13,C12,C11)	21.27	D(C17,C13,C12,C0)	21.71
D(C23,C14,C12,C11)	25.89	D(C17,C13,C12,C11)	24.84
D(C23,C14,C12,C13)	28.23	D(C18,C13,C12,C11)	20.06
D(C50,C46,C37,C36)	26.48	D(C23,C14,C12,C13)	37.39
D(C51,C46,C37,C36)	23.28	D(C24,C14,C12,C11)	-44.35
D(C60,C55,C48,C49)	20.09	D(C24,C14,C12,C13)	44.9
D(H77,C25,C15,C16)	69.53	D(C51,C46,C37,C33)	-24.48
D(H77,C25,C15,C19)	-48.8	D(C59,C55,C48,C47)	-21.76
D(H78,C25,C15,C16)	-68.63	D(C60,C55,C48,C47)	22.24
D(H78,C25,C15,C19)	-64.84	D(H78,C25,C15,C19)	-48.55
D(H79,C26,C21,C20)	20.69	D(H81,C26,C21,C22)	22.11
D(H79,C26,C21,C22)	-21.48	nan	NaN
D(H81,C26,C21,C20)	-23.57	nan	NaN

Table S5: Changes of bond length in angstroms, angle and dihedral angle in degrees for C257 during relaxation from ground state to excited state (ΔE) and cationic state (ΔC). Change in bond length, bond angle or dihedral angle connecting donor and acceptor is highlighted by green box.

Definition	ΔE	Definition	ΔC
B(C6,C4)	0.031	B(C17,C16)	-0.022
B(C7,C6)	0.02	B(C22,C21)	-0.02
B(C16,C15)	0.0248	B(C28,C26)	0.0261
B(C17,C16)	-0.0268	B(C31,N29)	0.0326
B(C23,C22)	0.0285	B(C49,C0)	0.0276
B(C30,N29)	0.0292	B(N29,C28)	-0.0254
B(N29,C28)	-0.0636		
B(O43,C39)	-0.0202		
B(S52,C6)	-0.021		
A(N29,C30,C32)	2.55	A(C4,C6,C7)	3.11
		A(C4,C6,S52)	3.59
		A(C20,C22,C23)	-3.4
		A(C26,C28,C27)	-2.73
		A(C28,N29,C30)	-3.32
		A(C28,N29,C31)	4.71
		A(C30,N29,C31)	-6.15
		A(O50,C49,O51)	-2.51
D(C7,C6,C4,C3)	34.43	D(C7,C6,C4,C3)	32.01
D(C18,C17,C16,C15)	20.54	D(C7,C6,C4,C5)	36.05
D(C25,C23,C22,C20)	20.71	D(C18,C17,C16,S53)	21.21
D(C31,N29,C28,C27)	-46.22	D(C19,C17,C16,S53)	21.46
D(C32,C30,N29,C28)	21.31	D(C30,N29,C28,C26)	48.5
D(C33,C30,N29,C28)	23.56	D(C30,N29,C28,C27)	-49.1
D(C33,C30,N29,C31)	-22.83	D(C31,N29,C28,C26)	-45.61
D(S52,C6,C4,C3)	-24.42	D(C31,N29,C28,C27)	-44.4
		D(C41,C31,N29,C28)	29.2
		D(C41,C31,N29,C30)	-25.42
		D(S52,C6,C4,C3)	-33.74

Table S6: Changes of bond length in angstroms, angle and dihedral angle in degrees for D5L2A1 during relaxation from ground state to excited state (ΔE) and cationic state (ΔC). Change in bond length, bond angle or dihedral angle connecting donor and π-spacer is highlighted by blue box and connecting structural parameters between π-spacer and acceptor is highlighted by red box.

Definition	ΔE	Definition	ΔC
B(C13,N6)	-0.0388	B(C10,N6)	0.0262
B(C19,C2)	0.0241	B(C13,N6)	-0.0336
B(C24,C23)	-0.0207	B(C26,C24)	0.0267
B(C28,C27)	0.0208	B(C27,C26)	-0.0366
B(N6,C5)	0.0793	B(N6,C5)	0.0508
		B(S25,C24)	-0.0221
A(C10,N6,C13)	-4.99	A(C5,N6,C10)	3.95
D(C11,C10,C9,C8)	-46.92	D(C10,N6,C5,C4)	-46.49
D(C13,N6,C5,C4)	47.72	D(C11,C10,C9,C8)	-49.9
D(C14,C13,N6,C5)	47.7	D(C14,C13,N6,C5)	43.77
D(C14,C13,N6,C10)	-27.91	D(C15,C14,C13,C18)	30.97
D(C18,C17,C16,C15)	-34.81	D(C18,C13,N6,C10)	31.09

Table S7: Changes of bond length in angstroms, angle and dihedral angle in degrees for D5L2A3 during relaxation from ground state to excited state (ΔE) and cationic state (ΔC). Change in bond length, bond angle or dihedral angle connecting donor and π-spacer is highlighted by blue box and connecting structural parameters between π-spacer and acceptor is highlighted by red box.

Definition	ΔE	Definition	ΔC
B(C5,C4)	-0.0205	B(C19,C2)	0.0233
B(C10,N6)	0.0233	B(C29,S27)	0.0226
B(C13,N6)	0.029	B(C34,C24)	0.029
B(C21,C20)	0.0273	B(N6,C5)	0.0558
B(C28,C26)	0.0428		
B(C29,S27)	0.0292		
B(C34,C26)	-0.0361		
A(C10,N6,C13)	-3.2	A(C23,C24,C34)	-3.34
A(C23,C24,C34)	-2.59	A(C28,C26,C34)	-2.57
A(C28,C26,C34)	-2.82	A(S27,C26,C34)	2.79
A(S27,C26,C34)	2.88		

References

(1) Kakiage, K.; Aoyama, Y.; Yano, T.; Otsuka, T.; Kyomen, T.; Unno, M.; Hanaya, M. An achievement of over 12 percent efficiency in an organic dye-sensitized solar cell. *Chem. Commun.* **2014**, *50*, 6379–6381.

(2) Sun, Z.-Z.; Geng, X.-J.; Ding, W.-L.; Feng, S.; Xu, Y.-L.; Li, Y.-L.; Xiao, Z.-Y.; Sun, S.-J.; Zhao, Z.-Q.; Liu, J.-F. Rational tailoring of C275 towards promising organic dyes: Enhancing light absorption and charge separation. *Synth. Met.* **2018**, *237*, 1–9.

(3) Zhang, M.; Wang, Y.; Xu, M.; Ma, W.; Li, R.; Wang, P. Design of high-efficiency organic dyes for titania solar cells based on the chromophoric core of cyclopentadithiophene-benzothiadiazole. *Energy Environ. Sci.* **2013**, *6*, 2944–2949.

(4) Liang, J.; Zhu, C.; Cao, Z. Electronic and optical properties of the triphenylamine-based organic dye sensitized TiO 2 semiconductor: insight from first principles calculations. *Phys. Chem. Chem. Phys.* **2013**, *15*, 13844–13851.

(5) Liao, J.; Zhao, H.; Xu, Y.; Zhou, W.; Peng, F.; Wang, Y.; Fang, Y. Novel BODIPY dyes with electron donor variety for dye-sensitized solar cells. *RSC Adv.* **2017**, *7*, 33975–33985.
